# What Chauncey Depew Says

**Published:** 1884-09

**Authors:** 


Chauncey Depew says the sooner a poor doctor, lawyer, or clergymen recognizes that his genius is for merchandize or types, or the skilled trades or accounts, the better for himself, his profession, and the world. He has secured positions for two lawyers —one as a brakeman, the other as a freight clerk—and both are advancing with rapid strides and confident aspirations toward the presidency of the road.

---

* The formula for Witzel's carbolic acid cement is as follows:

℞ Acid carbol., 5.0;
Alcohol absol, 2.0;
Aq. dist., 40.0;
Glycerinæ, 20.0.

Mix, and add an equal volume of the zinc chloride used in preparing oxychloride fillings. To a sufficient quantity of this mixture, add oxide of zinc, to give it the necessary consistence for filling.

